# P-1887. Outpatient vancomycin therapy: Rates of acute kidney injury in patients receiving area-under-the-curve-guided dosing as an inpatient followed by transition to trough-guided dosing after discharge

**DOI:** 10.1093/ofid/ofae631.2048

**Published:** 2025-01-29

**Authors:** Benjamin Honan, Lindsay Lim, Nicole M Hall, Janet Kim, Daniela F De Lima

**Affiliations:** University of Alabama at Birmingham Heersink School of Medicine, Birmingham, Alabama; University of Alabama at Birmingham, AdventHealth, Orlando, Florida; UAB Hospital, Birmingham, Alabama; Division of Infectious Diseases, University of Alabama at Birmingham Heersink School of Medicine, Birmingham, AL USA, Birmingham, Alabama; University of Alabama in Birmingham, Vestavia hills, Alabama

## Abstract

**Background:**

Vancomycin is one of the most prescribed antibiotics for hospitalized patients. Targeting an area under the curve to a minimum inhibitory concentration (AUC/MIC) ratio of 400-600 mg*hour/L has been associated with a reduction in vancomycin-induced acute kidney injury (AKI) compared to trough-guided dosing. Data evaluating AUC-guided vancomycin dosing in outpatient parenteral antimicrobial therapy (OPAT) are limited, and resources required for AUC-guided dosing are often unavailable. However, trough-guided dosing has been associated with vancomycin overexposure and in the outpatient setting, rates of AKI as high as 28.5% have been reported. We aim to evaluate outcomes of patients discharged on at least one week of vancomycin on a dose established through AUC-guided dosing during hospitalization followed by trough-guided dosing in the outpatient setting.

**Figure:**
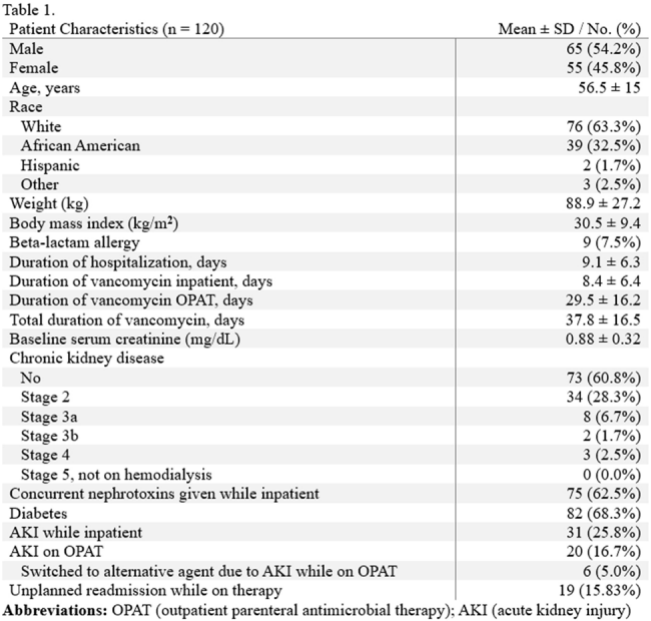

**Methods:**

In this retrospective single-center cohort study, we identified 120 patients admitted to the University of Alabama (UAB) Hospital or UAB Hospital-Highlands between October 1, 2021, and March 31, 2022, who met criteria for inclusion. Though we collected additional demographics and clinical information, our primary outcome was the development of AKI, as defined by the 2012 KDIGO guidelines, while receiving vancomycin via OPAT.

**Figure:**
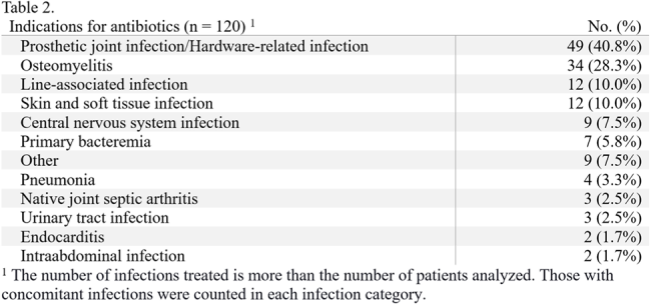

**Results:**

Approximately 17% of patients developed an AKI using this hybrid model. The most treated infection was prosthetic joint or hardware-associated infections (40.8%), and the most targeted organism was MRSA (41.7%). Vancomycin monotherapy comprised almost half of the antibiotic regimens on discharge (48.3%); the second agent, if prescribed, was typically a beta-lactam other than piperacillin-tazobactam (36.7%).

**Figure:**
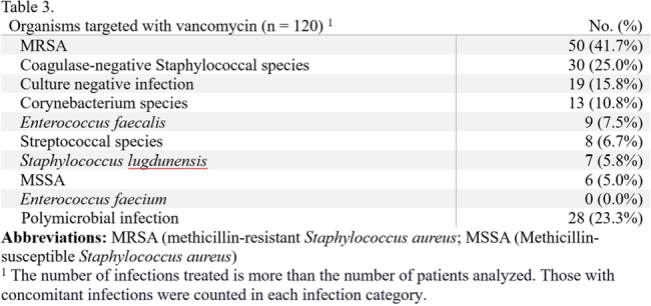

**Conclusion:**

Future directions include identifying predictors for the development of AKI while receiving vancomycin via OPAT and comparing outcomes of this hybrid model with a historical cohort of patients at our institution who received trough-guided dosing in both the inpatient and outpatient settings. Given the paucity of data on vancomycin dosing in the outpatient setting, investigating optimal and sustainable OPAT vancomycin dosing and monitoring is of the utmost importance to minimize adverse events and maximize patient safety.

**Figure:**
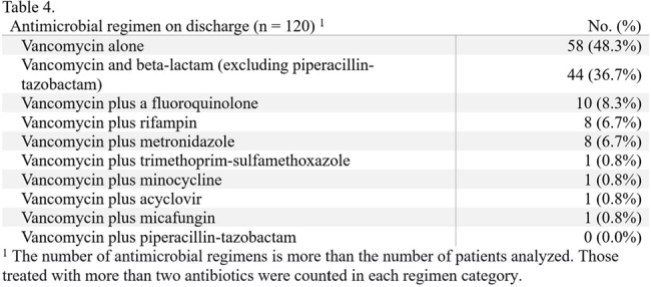

**Disclosures:**

All Authors: No reported disclosures

